# Protease activated receptors 1 and 4 sensitize TRPV1 in nociceptive neurones

**DOI:** 10.1186/1744-8069-6-61

**Published:** 2010-09-27

**Authors:** Vittorio Vellani, Anna M Kinsey, Massimiliano Prandini, Sabine C Hechtfischer, Peter Reeh, Pier C Magherini, Chiara Giacomoni, Peter A McNaughton

**Affiliations:** 1 Department of Pharmacology, University of Cambridge, Tennis Court Road, Cambridge CB2 1PD, UK; 2 Dipartimento di Scienze Biomediche, via Campi 287, Università degli Studi di Modena e Reggio Emilia, I-41100 Modena Italy; 3 Department of Physiology and Experimental Pathophysiology, University of Erlangen-Nürnberg, Universitätsstrasse 17, D-91054 Erlangen, Germany; 4 Università degli Studi della Repubblica di San Marino, Dipartimento di Economia e Tecnologia, Strada della Bandirola, 44, 47898 Montegiardino - Repubblica di San Marino - RSM

## Abstract

Protease-activated receptors (PAR1-4) are activated by proteases released by cell damage or blood clotting, and are known to be involved in promoting pain and hyperalgesia. Previous studies have shown that PAR2 receptors enhance activation of TRPV1 but the role of other PARs is less clear. In this paper we investigate the expression and function of the PAR1, 3 and 4 thrombin-activated receptors in sensory neurones. Immunocytochemistry and in situ hybridization show that PAR1 and PAR4 are expressed in 10 - 15% of neurons, distributed across all size classes. Thrombin or a specific PAR1 or PAR4 activating peptide (PAR1/4-AP) caused functional effects characteristic of activation of the PLCβ/PKC pathway: intracellular calcium release, sensitisation of TRPV1, and translocation of the epsilon isoform of PKC (PKCε) to the neuronal cell membrane. Sensitisation of TRPV1 was significantly reduced by PKC inhibitors. Neurons responding to thrombin or PAR1-AP were either small nociceptive neurones of the peptidergic subclass, or larger neurones which expressed markers for myelinated fibres. Sequential application of PAR1-AP and PAR4-AP showed that PAR4 is expressed in a subset of the PAR1-expressing neurons. Calcium responses to PAR2-AP were by contrast seen in a distinct population of small IB4^+ ^nociceptive neurones. PAR3 appears to be non-functional in sensory neurones. In a skin-nerve preparation the release of the neuropeptide CGRP by heat was potentiated by PAR1-AP. Culture with nerve growth factor (NGF) increased the proportion of thrombin-responsive neurons in the IB4^- ^population, while glial-derived neurotropic factor (GDNF) and neurturin upregulated the proportion of thrombin-responsive neurons in the IB4^+ ^population. We conclude that PAR1 and PAR4 are functionally expressed in large myelinated fibre neurons, and are also expressed in small nociceptors of the peptidergic subclass, where they are able to potentiate TRPV1 activity.

## Introduction

Proteases released during injury activate protease-activated receptors (PARs), a family of four G protein-coupled receptors, by cleaving the extracellular N-terminal domain to expose a tethered peptide ligand [1-5]. PAR1, PAR3, and PAR4 are activated by thrombin, reviewed in [5,6], while PAR2 is not activated by thrombin but is activated by trypsin and mast cell tryptase [7-9]. PAR4 is specifically activated by cathepsin G [10].

In sensory neurons of the dorsal root ganglia (DRG) a functional response to thrombin was initially reported by Gill et al [11]. The mRNA of all four PARs is expressed in sensory neurons [12]. There is clear evidence for the functional involvement of PAR2 receptors in peripheral mechanisms of inflammation and pain [13-15], partly via sensitisation of the transient receptor potential vanilloid subfamily 1 (TRPV1) receptor [15-18] and partly by stimulating the release of substance P and CGRP from the terminals of afferent neurons [13,19,20]. Sensitization of TRPV1 depends on activation of the epsilon isoform of PKC (PKCε), which can be observed as a translocation of PKCε from the cytoplasm to the surface membrane [21], and a similar translocation has been reported in response to activation of PAR2 [22].

Thrombin is released by blood clotting following blood vessel damage or tissue injury, and can act on PAR1, 3 and 4 expressed in primary sensory nerve terminals present in the vicinity. Thrombin injected into peripheral tissues induces proinflammatory effects, such as protein extravasation and vasodilation, which are mediated at least in part by a neurogenic mechanism [9,14,23]. Activation of PAR1 may be involved in peripheral nerve damage [24,25]. Some reports, however, describe antinociceptive effects of activation of peripheral PAR1 activation with subinflammatory protease concentrations [26,27]. PAR4 activation has also been shown to be analgesic [28-30], but other evidence shows that the administration of a PAR4 activator peptide (PAR4-AP) causes the formation of edema and leukocyte recruitment in a rat paw model of inflammation [31].

To the best of our knowledge no studies have investigated the localization of functional PAR1, 3 and 4 receptors in sensory neurons, nor the role of receptors activated by thrombin in TRPV1 sensitisation or in activation of PKCε in nociceptors. These questions are addressed in the present study. We initially compared the effects of thrombin in adult and neonatal rats and mice in order to compare PAR functional expression in different species and ages. In fact, though, we saw few qualitative or quantitative differences between these four groups of animals in responses to PAR activation. Most experiments were therefore continued in neurons from adult mice only, which also gave us the opportunity to compare the results in wild-type and transgenic animals in which the roles of specific PAR receptors were explored by deletion of PAR1 or PAR2.

## Methods

### Culture of dissociated DRG neurones

DRGs were removed from adult and neonatal rats (Sprague-Dawley,) or adult and neonatal C57BL/6J mice (neonatal rats and mice were both day 5-10 after birth). For experiments on the effects of gene deletion PAR1 (PAR1^-/-^), PAR2-deficient (PAR2^-/-^) and wildtype mice were bred from the descendants of littermates from heterozygous crosses (genetic background: C57BL/6 strain) originally obtained from Charles River Laboratories (Toulouse, France) and kindly made available to us by Prof. M. Steinhoff. DRGs were incubated in 0.25% collagenase (type IV, Worthington, Reading, UK) in PBS followed by mechanical trituration. Cells were centrifuged and resuspended in culture medium (DMEM containing 10% FBS, 1% penicillin/streptomycin solution and 1% L-glutamine, GIBCO), 10 μM cytosine arabinoside (Sigma) and, where appropriate, 50 ng/ml nerve growth factor (Promega) or 100 ng/ml neurturin or GDNF (Peprotech). Neurones were plated onto glass coverslips (BDH, UK), coated with 10 μg/ml poly-L-lysine (Sigma, UK) and 5 μg/ml laminin (BD Biosciences, Cowley, UK).

### Electrophysiology

Methods used were as described before [32-34]. In brief, all recordings were made from the somata of DRG neurons with the whole cell patch-clamp method, at a holding potential of -70 mV, using an Axopatch 200B amplifier and pClamp software (Molecular Devices, Palo Alto, CA). Test solutions were applied using a multibarrel automated rapid solution changer (CVscientific, University of Modena, Modena, Italy). Only one recording was performed on each culture dish to ensure that data were not obtained from cells that had been inadvertently exposed to other test treatments. All experiments were performed at room temperature (20 -22°C).

### Immunohistochemistry: DRG sections

**Figure 1:**
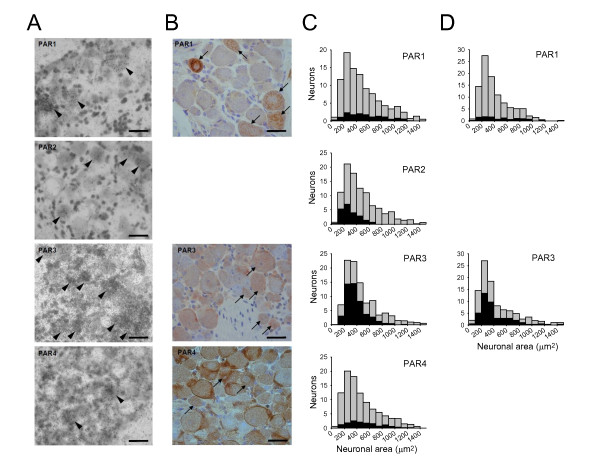
**Expression of PAR1-4 in sections of adult mouse DRG**. A. *In situ *hybridisation (ISH) for PAR1-4 carried out as described in Methods. Positive cells shown by arrowheads. Sections counterstained using hematoxylin-eosin. Scale bars 40 μm. B. Similar sections in which PAR1, 3 and 4 expression was determined using immunohistochemistry. Positive cells shown with arrows. The PAR2 antibodies available to us were found to be non-specific on Western blots and results for PAR2 are therefore not shown. Sections counterstained using hematoxylin-eosin. Scale bars 40 μm. C. Expression of PAR1 - 4 as a function of neuronal size in adult mouse DRG using ISH. Overall neuronal population (grey) is compared with those positive for each PAR isoform (black). Overall, PAR1 was found to be expressed in 15.0% of neurones, PAR2 in 21.5%, PAR3 in 49.5% and PAR4 in 14.5%. D. Similar results obtained using immunohistochemistry. PAR2 is not shown because the antibody was found to exhibit non-specific binding, and PAR4 is not shown because it proved impossible to distinguish neuronal from glial cell staining (see B). Overall PAR1 was expressed in 10.3% of neurones, and PAR3 in 42.0%.

DRGs from adult male TO mice (25-30 g, Tucks, UK) were rapidly removed and post-fixed in 10% formalin solution for 72 h, embedded in paraffin, sectioned at 4 μm on a sledge microtome (Leitz, Nussloch, Germany) and mounted on Fisher Superfrost/Plus slides (BDH, UK). Sections were dewaxed in xylene, incubated in 0.3% hydrogen peroxide in methanol to quench endogenous peroxidase activity and hydrated through an ethanol series. Sections were then blocked in 5% normal goat serum in 0.01 M PBS containing 0.03% Triton X-100, prior to overnight incubation at +4°C with the respective antibodies. Affinity-purified goat polyclononal IgGs (concentration 200 μg/ml) were obtained from Santa Cruz Biotechnology Inc (California, USA) and had been previously characterised by Western blot and immunohistochemistry. Anti-PAR1 (sc-8204; 1/100 dilution) was raised against a peptide mapping at the N-terminal of mouse PAR1, and reacts with PAR1 of mouse and rat origin. Anti-PAR3 (sc-8209; 1/800) was raised against a peptide mapping to the C-terminus of mouse PAR3, and reacts with PAR3 of mouse and rat origin. Anti-PAR4 (sc-8462; 1/150) was raised against a peptide mapping at the C-terminal of PAR4 of mouse origin, and reacts with PAR4 of mouse and rat origin. Immunoreactivity was detected using biotinylated donkey anti-goat secondary antibodies raised against the primary antibody host (7.5 μg/ml, Vector Laboratories, Peterborough, UK), followed by avidin-biotin complex (ABC) (Vector Laboratories, UK) and subsequently visualised using diaminobenzidine (DAB)/hydrogen peroxide (Biogenex, Finchampstead, UK). All immunohistochemical detection steps (from secondary antibody stage onwards) were performed on an Optimax (Biogenex, UK) robotic immunostainer to increase intersection staining consistency, thereby increasing the accuracy and reliability of semiquantitative analysis. Sections were counterstained with Gill's haematoxylin followed by acid-alcohol (0.5% concentrated hydrochloric acid in 70% ethanol). Control experiments for immunohistochemistry were performed by incubation with normal goat serum in place of primary antibodies and resulted in a complete absence of staining (not shown). Specific labelling was tested by incubation of sections with affinity-purified antisera and a 20-fold excess of peptide obtained from Santa Cruz Biotechnology Inc (California, USA) and corresponding to the antigenic sequence to which the antisera were raised. Antibody blocking in this way resulted in a complete absence of specific staining (not shown), though background levels were similar to those shown in Fig. 1.

### In situ hybridisation (ISH)

The DRGs of 25-30 g adult male TO mice were rapidly removed after cervical dislocation, frozen in isopentane, chilled to -40°C and sectioned at 10 μm using a Brights cryostat (model OTF). Sections were post-fixed with 4% paraformaldehyde in PBS, pH 7.2, dehydrated through an ethanol series and stored in 95% ethanol at 4°C until use. Oligonucleotide probes specific to mouse PARs were designed (see Table 1) and custom-synthesised by Sigma Genosys (Cambridge, UK). Purification was by 8M urea/8M polyacrylamide preparative sequencing gel electrophoresis. Specificity was thoroughly checked using BLAST. For PAR1, the probe was synthesised complementary to bases 969-1008 (according to GenBank Acc. No L03529); for PAR2, complementary to bases 930-969, according to [35]; for PAR3, complementary to bases 351-390, according to [36]; and for PAR4, complementary to bases 1059-1098, according to [37,38].

**Table 1:** Oligonucleotide probe sequences for in situ hybridisation studies

Receptor	Oligonucleotide probe (5'→3')	Probe length
PAR1	caaagcagacgatgaagatgcagaacaccgcggcagacag	40
PAR2	gaagtacatggccagcacggtgatgatgagtcggatagcc	40
PAR3	tcacgtggagagttgaaatactgtcctcgggacactccgc	40
PAR4	catagcgcgtaccttctccctgaactcatgggacacatag	40

Probes were 3'-end-labelled using TdT-mediated addition of [^35^S]deoxyadenosine 5' α-thiotriphosphate (NEN, Hounslow, UK) [39]. Hybridisation was carried out as previously described [40]. Briefly, 3.5 × 10^5 ^cpm of ^35^S-labelled probe in 100 μl of minimalist hybridisation buffer (50% deionised formamide, 4 × standard saline citrate [SSC], 10% dextran sulphate, and 40 mM DTT, all from Sigma except dextran sulphate, Anachem, Luton, UK) was placed on each slide and coverslipped with a Parafilm (Sigma, Poole, UK) coverslip. Hybridisation was carried out overnight in a humid environment at +39°C. To define non-specific hybridisation, adjacent sections were incubated in a mixture of labelled probe with excess (100-fold) of unlabelled probe. In all cases, incubation of sections with 100-fold excess of unlabelled 'cold' probe abolished the signal. Hybridised probes were detected at the cellular level using NTB2 nuclear emulsion (Kodak, Hemel Hempstead, UK). Sections were counterstained with Gill's haematoxylin and eosin Y alcoholic solution.

The cross sectional areas of neuronal profiles with a visible nucleus were measured using the Scion Image Analysis system. Silver grains overlying each identified neuronal profile were counted for PAR1, PAR2 and PAR4. For PAR3 the high levels of expression of this receptor's mRNA made grain counting impossible, and cells were given a score (-, negative/below detectable levels; +, weakly labelled; ++ moderately labelled; +++, intensely labelled). Signal intensity for PAR1, PAR2 and PAR4 was determined by dividing grain counts by the area of the neuronal profile. To reduce the risk of biased sampling of the data owing to varying emulsion thickness and background density of silver grains for each section, a signal/noise (S/N) ratio was used, as described previously [40]. The signal intensity of each neuronal profile was expressed as a S/N ratio of the mean background level as described [41,42]. Neuronal signal intensities greater or equal to three times the background level (S/N≥3) were considered positively labelled.

### Immunocytochemistry: isolated DRG neurons and glia

For PKCε visualization, rat DRG neurons cultured for 1-3 d in vitro were treated with a PAR agonist (for times see Table 2) and then rapidly fixed for 10 min at room temperature with paraformaldehyde/PBS (4% formaldehyde and 4% sucrose mixed 50:50 with PBS). Fixed cells were washed three times in PBS (with 0.1% fish skin gelatin to block nonspecific binding), permeabilized for 30 min at room temperature with Triton X-100 (0.2% in PBS), and incubated overnight at 4°C with rabbit polyclonal anti-PKCε antibody [33] diluted 1:1000 in PBS-T/gelatin (PBS with 0.05% Triton X-100). Coverslips were then incubated for 1 h at room temperature with donkey anti-rabbit IgG conjugated to the fluorophore Alexa Fluor 488 (1:200; Invitrogen), washed three times in PBS/gelatin, and visualized with a confocal microscope (Leica SP2).

**Table 2:** PAR activators and concentrations used

PAR activator	Concentration	Selective for	Source
Thrombin	0.01-100 nM	PAR1,3,4	Sigma, cat T4648
Cathepsin G	1-1000 nM	PAR4	Sigma, cat C4428
Trypsin	0.1-10 μM	PAR1,2,3,4	Sigma, cat T9201
Collagenase	0.25%	-	Sigma, cat 4188
type IV			
TFLLR-NH_2_	100 μM	PAR1	Tocris, cat 1464
SFLLRN-OH (for rat)	100 μM	PAR1	Bachem, cat H8365
SLIGRL-NH_2_	100 μM	PAR2	Tocris, cat 1468
AYPGKF-NH_2_	200 μM	PAR4	Sigma, cat A3227

To characterize subpopulations of protease-responsive neurons, double immunostaining on DRG cultures was performed. Coverslips processed for PKCε immunoreactivity as above were incubated overnight at 4°C with the following polyclonal antibodies: anti-substance P (SP), anti-calcitonin gene-related peptide (CGRP), anti-N52 (1:100; all polyclonal antibodies from Santa Cruz Biotechnology, CA), anti-parvalbumin (1:1000, Sigma) or anti-COX-1 (1:100, Cayman Chemicals, cat. no. 160110). After washing, coverslips were exposed for 1 h at room temperature to donkey anti-goat antibodies (or goat anti-mouse for anti-parvalbumin and anti-COX-1 antibodies) conjugated to the fluorophore Alexa Fluor 594 (1:200, Invitrogen), washed three times in PBS/gelatin, and visualized. Double staining for IB4, on coverslips previously processed for PKCε, was assessed by incubating the cells for 1 h at room temperature with IB4 bound to Alexa Fluor 594 (1:100, Invitrogen) followed by washing (three times). Coverslips were stored at 4°C in sodium azide (0.05% in PBS) for additional analysis. Non-neuronal cells in DRG cultures were identified as glial by morphology by their lack of response to 25 mM KCL and by staining with the glial-specific anti-S100 antibody (Sigma) (not shown).

### Quantification of PKCε translocation

Activation of PKCε results in translocation from an entirely cytoplasmic location to the neuronal cell membrane. Translocation was quantified by determining fluorescence intensity along a line positioned across the cell so as to avoid the nucleus (for details see Cesare et al, 1999). Neurones in which intensity at the cell membrane was 1.5× greater than the mean of cytoplasmic intensity were counted as positive.

### Intracellular calcium imaging

Calcium imaging was performed as described previously [32-34]. In brief, isolated DRG neurons, plated onto glass coverslips were loaded with the calcium-sensitive fluorescent indicator Fluo-4 AM (10 μM; Invitrogen). Coverslips were imaged with an inverted confocal microscope (MicroRadiance; Bio-Rad, Hemel Hempstead, UK) or with a camera-based system (Andor Technology, Belfast, UK) in HBSS (140 mM NaCl, 1.8 mM CaCl_2_, 1 mM MgCl_2_, 4 mM KCl, 10 mM HEPES, 4 mM glucose, pH 7.4). High numerical aperture 10× or 20× objectives were used. PAR agonists used are given in Table 2 below. The peptide agonist TFLLR was used to activate PAR1 apart from in experiments on intact rat skin, where the more effective rat agonist SFLLRN was used. Proteases were purchased as 1,000 NIH units and concentrations were calculated from conversion factors supplied by the manufacturer giving units/mg protein. Neurons were distinguished from non-neuronal cells by applying 25 mM KCl, which induces a rapid increase of [Ca^2+^]_i _only in neurons. At the end of the experiment, the maximal fluorescence (F_max_) was obtained by application of ionomycin (10 μM; Calbiochem, La Jolla, CA) in the presence of Ca^2+ ^(30 mM) and K^+ ^(125 mM). Data are expressed as ΔF/F_max_. All experiments were performed at 20-22°C (RT).

The protocol used for sensitisation experiments was similar to that previously described [43]. In brief, cells were exposed to short (1.6 s) repeated capsaicin applications, and PAR activator peptides or proteases (Table 2) were applied for 2 min prior to the sixth capsaicin application. For control experiments PAR activator application was omitted. The ratios obtained by dividing the amplitude of the sixth capsaicin peak by the amplitude of the fifth capsaicin peak were used to plot a histogram for each treatment group. The distribution seen for each group was compared with the distribution obtained from control, vehicle-treated cells. A neuron was defined as sensitized if the ratio was greater than the upper 99.7% confidence interval calculated from control neurones. Ad-hoc software was written for analysis of calcium imaging traces.

### CGRP release studies

Male Wistar rats (80 - 100 g) were sacrificed in CO_2 _and the hairy skin of the hindpaw (26 ± 14 g, mean ± SD) was subcutaneously excised from the knee to the foot sparing larger vessels and nerves. The skin flap was wrapped around an acrylic rod with suture thread, exposing the corium side, and the preparation was placed in a beaker containing physiological buffer solution bubbled with 95% O_2_, 5% CO_2_, pH 7.4 in a water bath (32°C) for 30 min to equilibrate. The skin sample was then sequentially advanced in 5 min intervals through a series of six glass test tubes filled with 1.2 ml gassed buffer and mounted in a shaking bath. The first tube was to measure basal CGRP secretion, the subsequent three tubes (15 min) contained the PAR1 or PAR2 activator peptide (table 2) or buffer solution for control, the fifth tube was at a temperature of 47°C to apply noxious heat stimulation, and the sixth and final tube (again 32°C) was to measure recovery or residual CGRP release. Each incubation fluid was immediately processed to determine the CGRP concentration (pg/ml) using a commercial enzyme immunoassay kit according to the manufacturer's instructions (SPIbio, Montigny, France). The procedure has previously been validated and described in detail [44].

### Statistical analysis

Statistical comparisons were performed with one-way analysis of variance (ANOVA), followed by Bonferroni or Scheffé post hoc test and χ^2 ^(SPSS for windows); pairwise comparisons were made using Student's t-test.

## Results

### Expression of PARs in DRG neurons: in situ hybridisation

In situ hybridization (ISH) was implemented to determine the cellular distribution of PAR subtype mRNAs in DRGs from adult mice as previously described [40]. In Fig. 1A, bright-field photomicrographs of representative examples of autoradiographs show the localization of oligonucleotide probes complementary to mouse PAR1, 2, 3, 4 mRNA (arrowheads). Silver grains are visualised as small black dots overlying tissue sections. Probes with comparable activity were used for each receptor. PAR3 mRNA was intensely expressed in many DRG neurons and the mRNAs for PAR1, PAR2 and PAR4 were more weakly expressed. A comparison of expression intensity between receptor subtypes is subject to several variables, such as efficiency of the labelling reaction to incorporate 35S-dATP tails into the oligonucleotide probes, and hybridisation strength, though these effects were minimised as much as possible. However, within these limitations it seems clear that the expression of PAR3 is much more intense than that of PAR1, 2 and 4. In the case of PAR1, 2 and 4 a quantitative method was used to distinguish neurons with signal above background, see Methods and [40].

Fig. 1C shows histograms of cells positive for PAR1-4 against mean cross-sectional area of neuronal profiles (between 912 and 1072 cells obtained from five sections for each PAR and from three adult animals). Grey histograms indicate all neuronal profiles with nuclei present that were measured, and black bars show profiles with a positive in situ hybridization signal/noise ratio. PAR1 mRNA was found to be expressed in 15.0 ± 1.5% of DRG neurones across all size classes. PAR2 mRNA was present in 21.5 ± 3.4% of total neuronal profiles, almost exclusively in neurones with a small cross-sectional area; there was only a low level of mRNA expression in medium sized neurones and no detectable expression in neurones with a large cross-sectional area. PAR3 was present in 49.5 ± 4.5% of neurons and was expressed mainly in neurones with a small cross-sectional area, but unlike PAR2, PAR3 mRNA was also expressed in medium-sized neurones. PAR4 mRNA expression was found in a similar proportion of neurones to PAR1 (14.5% ± 4.3) and with a distribution of expression across the neuronal size range similar to that found for PAR1.

Expression of PARs in glia was difficult to detect unequivocally using ISH because of the small size of these cells and the scatter of silver grains. We show below that there is clear functional expression of PAR1 and PAR2 in glial cells.

### Expression of PARs in DRG neurons: immunohistochemistry

PAR1, 3 and 4 immunoreactivity (IR) was detected in DRG neurons (Fig. 1B). The PAR2 antibodies available to us showed clear evidence of non-specific bands on Western blot and results are therefore not shown. Preabsorption controls with a 20-fold excess of immunising peptide completely ablated the signals for PAR1, 3 and 4 in adjacent sections, as did incubation in the absence of primary antibody (data not shown). As with ISH, the distribution of expression was determined by measuring neuronal cross-sectional area, and by only including profiles in which there was a visible nucleus (Fig. 1D).

PAR1-IR was restricted to a small percentage of neurones (10.28 ± 2.54%) and was expressed in cells across the neuronal size range. The results for PAR1-IR were similar to those obtained with ISH (compare Fig. 1C and 1D). Expression was punctate and appeared particularly intense in vesicular structures surrounding the nucleus, suggesting the presence of large intracellular stores of protein. PAR3-IR was detected in 42.03 ± 4.95% of neuronal profiles (Fig. 1D), similar to results obtained using ISH (Fig. 1C). PAR4-IR was also detected in DRG neurons but PAR4-IR was particularly strongly expressed in glial cells, and it was not always possible to distinguish positive neurones stained at the plasma membrane from surrounding ensheathing glial cells (see Fig. 1B). For this reason PAR4-IR was not quantified.

### Calcium signals activated by PAR agonists in DRG neurons

**Figure 2:**
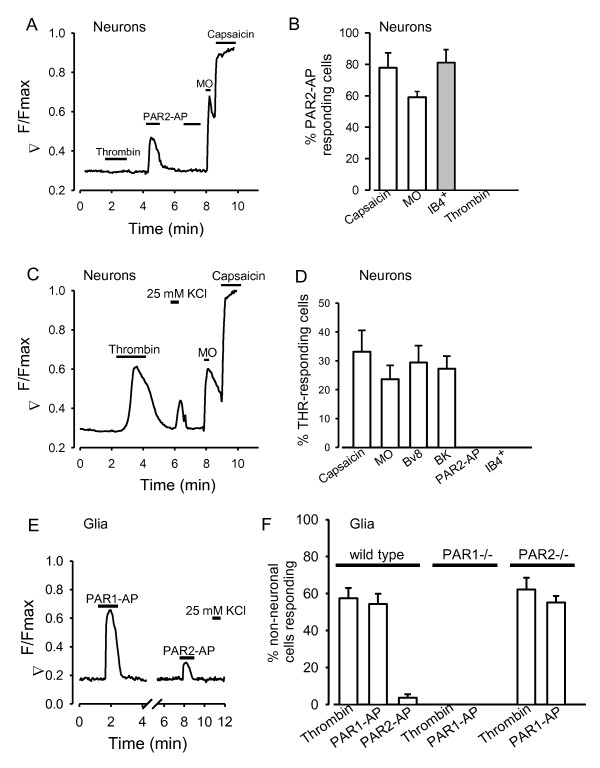
**Calcium signals elicited by PAR agonists**. A. Adult mouse neuron in which an increase in [Ca]_i _was elicited by a specific PAR2 activator peptide (PAR2-AP, SLIGRL, 100 μM), but not by thrombin (100 nM) which activates PAR1, 3 and 4. The neuron also expresses receptors for TRPA1 and TRPV1, as shown by its responses to the specific TRPA1 agonist mustard oil (MO, 100 μM) and the specific TRPV1 agonist capsaicin (1 μM). B. Most PAR2-AP-responsive adult mouse neurons also responded to capsaicin and mustard oil but none responded to thrombin (n = 180 neurones). Staining of unfixed cells with fluorescently labelled IB4 (isolectin B4 from Griffonia simplicifolia coupled to Alexa 594, Molecular Probes) immediately after the calcium imaging experiment showed that most PAR2-AP responsive neurons were IB4-positive (grey bar). Similar results were obtained in neonatal rat neurons (88.6 ± 5.1% of cells responding to PAR2-AP also responded to capsaicin, and 82.5 ± 6.0% were IB4^+^). C. Adult mouse neuron in which an increase in [Ca]_i _was elicited by thrombin (100 nM). This neuron also expresses the ion channels TRPA1 and TRPV1, as shown by its responses to mustard oil (MO, 100 μM) and capsaicin (1 μM). Cell was identified as a neuron on morphological grounds, confirmed by calcium increase observed in response to 25 mM KCl. D. Around 25-33% of thrombin-responsive neurons (n = 455) also responded to capsaicin (1 μM), mustard oil (100 μM), the peptide Bv8 (100 nM) and bradykinin (1 μM) but none responded to PAR2-AP (100 μM). Final bar shows that no thrombin-responsive adult mouse neuron bound IB4. E. Glial cell which responded with increase in [Ca]_i _to PAR1-AP (100 μM) and to PAR2-AP (100 μM). Cell was identified as a glial cell on morphological grounds, confirmed by absence of calcium increase in response to 25 mM KCl. In separate experiments, cells of this morphology were also identified by the glial-specific anti-S100 antibody (not shown). F. Percentage of glial cells responding to thrombin (100 nM), PAR1-AP (100 μM) and PAR2-AP (100 μM). Deletion of PAR1 ablated responses to both thrombin and PAR1-AP (bars 4 and 5) while deletion of PAR2 was without effect on responses to thrombin and PAR1-AP (bars 6 and 7).

We next examined functional activation of PAR receptors. A sub-population of small DRG neurons responded to the specific PAR2 activator peptide SLIGRL (PAR2-AP), which is derived from the activator domain of PAR2 (Fig. 2A). The proportion of neurons responding to the PAR2-AP with an increase in [Ca]_i _was 15.6% in neonatal rats and 12.1% in neurons from adult mice. A large majority of the PAR2-AP responsive neuronal population also expressed TRPV1 and TRPA1, as shown from the increase in [Ca]_i _in response to the specific TRPV1 agonist capsaicin and to the specific TRPA1 agonist mustard oil, and bound the plant isolectin B4 (IB4), which identifies a non-peptidergic subpopulation of nociceptors (see Fig. 2A and 2B). These PAR2^+ ^neurons therefore have the characteristics of IB4-positive nociceptors. None of these neurons, however, responded to thrombin and so are unlikely to express PAR1 or 4 (PAR3 appears unresponsive in DRG neurons, see below).

Thrombin, which activates PAR1, 3 and 4, elicited robust increases in [Ca]_i _in a distinct sub-population of sensory neurons (Fig. 2C,D). The proportion of neurons responding to thrombin with an increase in [Ca]_i _was 17.5% in neonatal rats and 15.2% in neurons from adult mice. No neuron responsive to thrombin also responded to PAR2-AP (Fig. 2B,D). Among these thrombin-responsive neurons, around 25-33% responded to capsaicin, mustard oil, and to the peptides Bv8 and bradykinin, both of which act on G-protein coupled receptors expressed in nociceptors [34], but none bound IB4 (Fig. 2D). About a third of thrombin-responsive neurons are therefore IB4-negative nociceptors, while the remainder are non-nociceptive. As PAR2 is predominantly expressed in IB4^+ ^nociceptors (see above) this shows that functional PAR2 and PAR1/3/4 receptors are located in separate subpopulations of nociceptors. PAR4 was found to be colocalised with PAR1 expression in neonatal rat neurons, because calcium responses to a PAR4-AP (AYPGKFR) were elicited in a subset of PAR1 expressing neurons (see below) and all cells responding to PAR4-AP also exhibited a calcium signal in response to PAR1-AP (not shown).

Glial cells are clearly distinguishable from neurons both on morphological grounds and because they do not exhibit a calcium increase in response to elevated [K^+^] (Fig. 2C). Most glial cells responded to thrombin (Fig. 2E). A few glial cells responding to thrombin also responded to PAR2-AP (3.6% -see Fig. 2E,F), showing that in contrast to neurons, PAR2 and PAR1/3/4 are co-expressed in a small subset of glial cells. The calcium response to thrombin and PAR1-AP in glial cells was ablated by genetic deletion of PAR1 but was unaffected by deletion of PAR2 (Fig. 2E).

**Figure 3:**
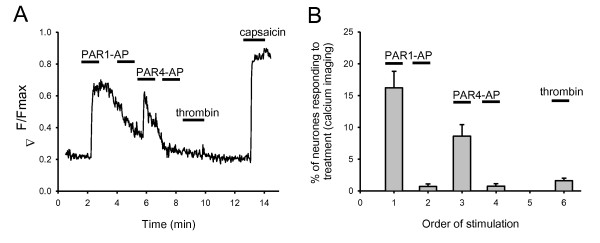
**Desensitization of PAR1 and PAR4 ablates calcium signals in response to thrombin**. A. Increase in [Ca]_i _recorded as in Fig. 2. Calcium increase elicited by application of PAR1-AP completely desensitizes response to a subsequent application of PAR1-AP but not to PAR4-AP. The calcium signal in response to thrombin was ablated in the large majority of cells by desensitization of both PAR1 and PAR4. All experimental details as in Fig. 2. B. Following desensitization of PAR1 and PAR4 only 1.6% of neurons gave a calcium signal in response to thrombin, compared with 15.2% in control neurons. Summary of results from n = 187 neurons from 4 separate coverslips.

PAR3 is highly expressed in DRG neurons (Fig. 1). PAR3 mRNA is seen in about 50% of neurons and PAR3 protein is seen in about 42% of neurons. Functional responses to thrombin, which should activate PAR3 (along with PAR1 and PAR4), are seen in a significantly lower number of neurons, however, suggesting that PAR3 is non-functional, at least when expressed in the absence of other PARs. To test this more conclusively it would be desirable to activate PAR3 alone, but specific activation of PAR3 in neurons coexpressing PAR1 and PAR4 is not possible because PAR3 peptides also activate PAR1 and PAR4 [45,46]. We therefore tested for PAR3 responses by desensitizing PAR1 and 4 with their specific activator peptides, and then retesting with thrombin (Fig. 3). Following desensitization of PAR1 and PAR4, calcium signals in response to thrombin are seen in only a very small number of neurons, far smaller than the proportion in which histological studies had shown expression of PAR3. These results support the idea the PAR3 is largely non functional by itself in DRG neurons, but they do not rule out the possibility that PAR3 may heteromerise with other PARs to form functional receptors, as has been found in other studies [47,48].

### Sensitization of TRPV1 by PAR activation

**Figure 4:**
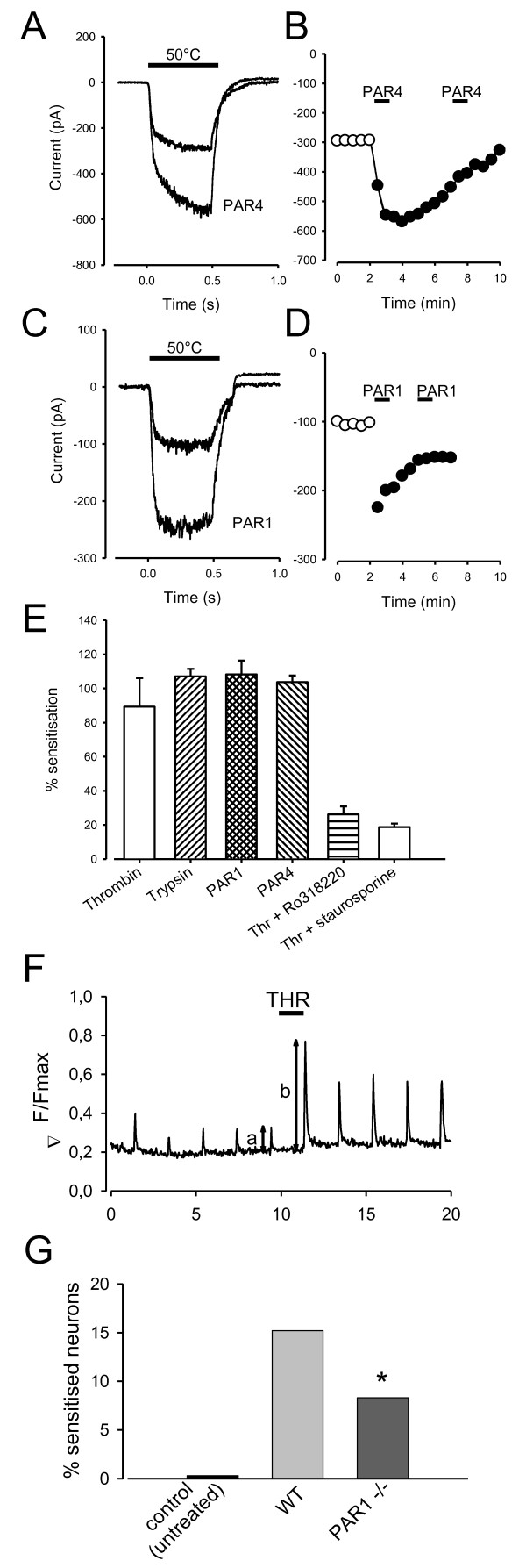
**Sensitization of TRPV1 by PAR activation**. A - D. Heat-activated currents were significantly enhanced in c. 10% of neurons by application of PAR1-AP and PAR4-AP. Single traces in panels to left are taken from time courses shown in right hand panels. Both PAR1-AP (TFLLR at 100 μM) and PAR4-AP (AYPGKF, 200 μM) caused long-lasting sensitisation. Sensitization showed complete tachyphylaxis on a second application. E. Percentage sensitization in experiments similar to those in A. Thrombin (100 nM), trypsin (100 nM), PAR1-AP and PAR4-AP all caused approximately a doubling of the inward current elicited by heat. Thrombin-induced sensitisation was largely blocked by the PKC inhibitor Ro318220 (1 μM) and by the broad-spectrum kinase inhibitor staurosporine (1 μM, both applied throughout the experiment). F, G Calcium imaging experiments to monitor functional sensitization of TRPV1 by thrombin. F shows typical experiment in which increases in [Ca]_i _elicited by successive brief exposures to capsaicin (500 nM, 1 s) were enhanced by exposure to thrombin (100 nM, black bar). All experiments performed in adult mouse neurones. G shows percentage of cells sensitized in experiments similar to those shown in F on neurons from WT and PAR1^-/- ^adult mice. Difference was significant (χ^2 ^test, *, p < 0.05).

Activation of the heat and capsaicin gated ion channel TRPV1 is potentiated by PAR2 activation [22]. Fig. 4 shows a similar potentiation of heat-activated inward currents by specific PAR1 and PAR4 activator peptides. Both PAR agonists caused substantial sensitization of TRPV1 in a subset of neurons (c. 10% of total neurons, consistent with studies of expression of PAR1 or PAR4, see above). Sensitization was long-lasting and subsequent PAR-AP applications were ineffective (Fig. 4B,D). Thrombin and trypsin also caused a substantial enhancement in the inward current activated by heat (Fig. 4E).

Many pro-inflammatory mediators sensitize TRPV1 via downstream activation of PKCε, reviewed in [49]. Consistent with this also being the principal signaling pathway activated by PAR1/3/4, Fig. 4E shows that the sensitization caused by thrombin was reduced at least 5-fold by the specific PKC inhibitor Ro-318220 or by the broad-spectrum kinase inhibitor staurosporine.

We next tested sensitization of TRPV1 by thrombin in wild-type and PAR1^-/- ^mice. In order to improve cell yield we employed a calcium imaging protocol similar to that used by Bonnington & McNaughton [43]. We activated TRPV1 by applying brief pulses of the specific agonist capsaicin, and tested the effect of thrombin in enhancing TRPV1 activation (Fig. 4F). Ratios of responses to capsaicin before and after application of thrombin were calculated, and sensitized cells were identified when the ratio exceeded the 99.7% confidence limits of a distribution obtained from control experiments (see Additional file 1). In PAR1^-/- ^animals the percentage of sensitized neurons using thrombin as a PAR activator was 8.3%, significantly lower than in WT neurons (Fig. 4G). Thus removal of PAR1 reduces but does not completely abolish the response to thrombin, consistent with the idea (see above) that DRG neurons also express functional PAR4 receptors.

Note that the percentage of neurons from PAR1^-/- ^mice responding to thrombin is similar to the proportion of neurons expressing PAR4 by ISH (14.5%, see Fig. 1C above) but is very much smaller than the proportion expressing PAR3 by both ISH and immunohistochemistry (49.5% and 42.03% respectively, see Fig. 1C,D above). Combined with the results in Fig. 3 (above) these results suggest that PAR4 receptors in DRG neurons are functionally activated by thrombin but that PAR3 receptors are not.

### PAR1/4 agonists cause translocation of PKC-ε in sensory neurons

**Figure 5:**
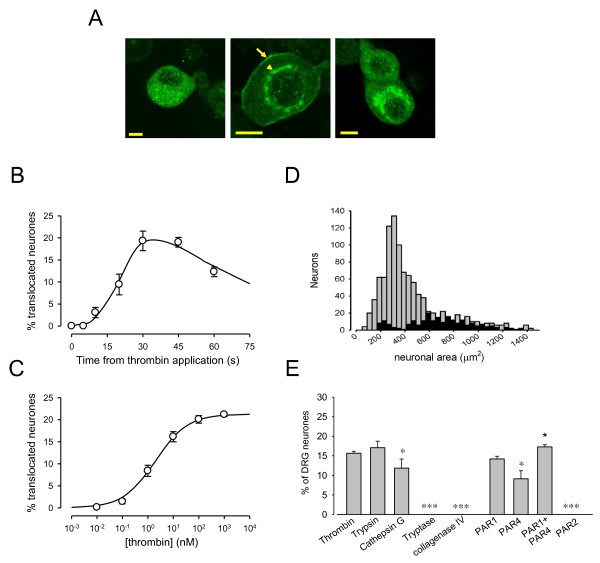
**Translocation of PKCε to neuronal surface membrane caused by thrombin**. A. Translocation of PKCε to neuronal surface membrane in control conditions (left) and following exposure to thrombin (100 nM, 30 and 60 sec). PKCε translocated rapidly to the surface membrane following application of thrombin (arrow in middle panel) and at longer times became progressively internalised (arrowhead in middle panel and right panel). Adult mouse neurons cultured in 10% FBS in absence of NGF and neurturin. Scale bars 5 μm. B. Percentage of neurons showing translocation to the plasma membrane as a function of time of exposure to thrombin (number of neurons > 2000 for each point). C. Peak percentage of neurons in which PKCε was translocated, as a function of thrombin concentration (number of neurons > 2000 for each point). Continuous curve shows a Hill equation with n = 0.7 and K_1/2 _= 2 nM. D. Size distribution of thrombin-responsive neurons. Grey bars show size distribution of overall neuronal population, and black bars show neurons in which PKCε translocation was observed following exposure to thrombin (100 nM, 30 s). E. Activation of PKCε translocation by proteases and specific PAR activator peptides. Thrombin, trypsin and cathepsin G (all 100 nM, 30 s) caused translocation of PKCε in a similar percentage of adult mouse neurons but tryptase and collagenase IV were ineffective. *, p < 0.05, ***, p < 0.001, t test compared to thrombin. PAR1-AP (TFLLR, 100 mM) caused translocation similar that of thrombin. PAR4-AP (AYPGKF, 200 mM, bar 2) caused translocation in a significantly smaller proportion of neurons when compared to PAR1-AP. Increased concentrations of activating peptides did not cause increased translocation (not shown). The effects of PAR1-AP and PAR4-AP (both at 100 mM) were partially but not completely additive. PAR2-AP (SLIGRL-NH_2_) had no effect. *, p < 0.05, ***, p < 0.001, t test compared to PAR1 alone.

The activation of PKCε can be visualized as a translocation from the cytoplasm to the cell surface membrane, and provides a sensitive indicator of those neurons activated by bradykinin [21,33] or by other pro-inflammatory mediators [34]. We found that thrombin and PAR1-AP caused a pronounced translocation of PKC-ε to the neuronal cell membrane in a subset of neurons from adult and neonatal rats and from adult mice (Fig. 5A). PKCε translocation, expressed as the percentage of neurons in which clear translocation was observed, peaked at 30 s after application of a maximal concentration of 100 nM thrombin (Fig. 5B). At longer application times PKCε was internalized into peri-nuclear vesicles (Fig. 5A, right hand panel), as is seen after longer exposures to bradykinin [21] and to the prokineticin receptor agonist Bv8 [34]. Translocation of PKCε was half-activated by a concentration of 2.0 ± 0.4 nM thrombin and was fully saturated at 100 nM thrombin (Fig. 5C). In adult mouse neurons cultured without NGF translocation was observed in 15.6 ± 0.5% of the population (Fig. 5D), a proportion which increased to 19.3 ± 1.0% with NGF (see below). Responsive neurons were distributed across all neuronal size classes, in agreement with histological data for expression of PAR1 and 4 (Fig. 1).

Other proteases known to activate PAR1 and PAR4 were also effective in causing translocation of PKCε (Fig. 5E). Trypsin, a broad-spectrum PAR activator, produced translocation in 17.1 ± 1.6% of neurons. Cathepsin G, which preferentially activates PAR4 over PAR1, caused translocation in 11.8 ± 2.3% of neurons. Type IV collagenase was ineffective. The PAR1-AP TFLLR caused translocation in a similar proportion of neurons to thrombin, while the specific PAR4-AP AYPGKF caused translocation in a significantly lower percentage of neurons than thrombin (9.1 ± 2.1%, n = 5 p < 0.05). Application of the PAR1-AP TFLLR in combination with PAR4-AP gave only a slightly higher percentage than PAR1-AP applied alone (17.3 ± 0.6%, n = 6). These data agree with those above (Fig. 3B) in showing that PAR4 receptors are expressed in a subset of the PAR1-expressing sensory neurons. PAR2-AP was ineffective in causing translocation of PKCε in any neuron.

### Characteristics of neurons expressing functional thrombin receptors

**Figure 6:**
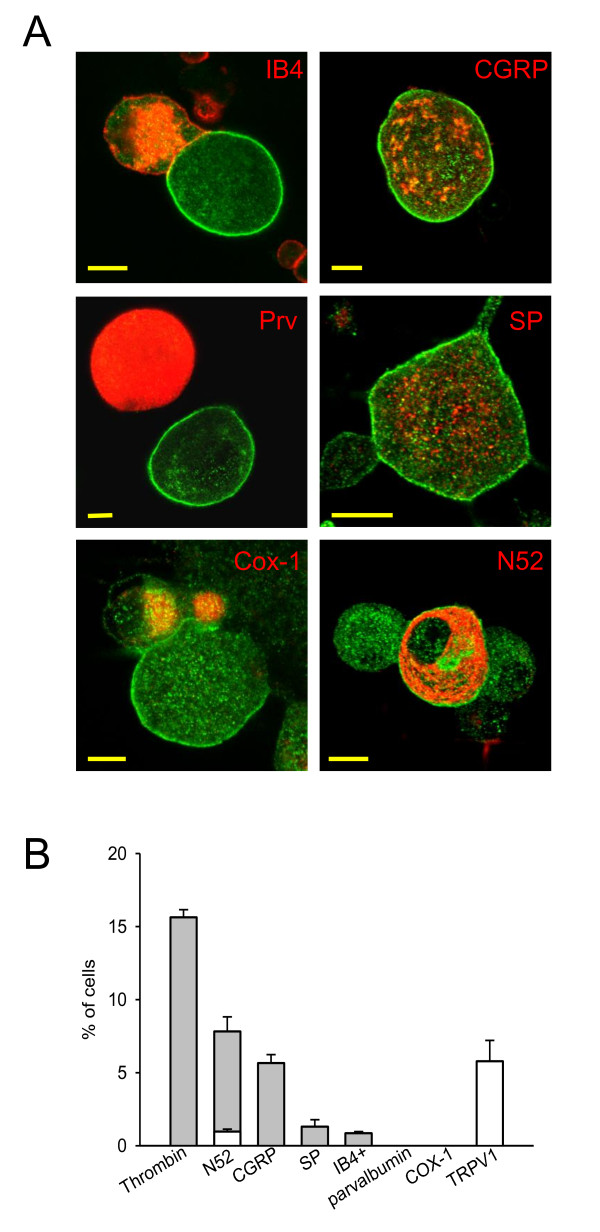
**Co-localisation of thrombin-induced translocation of PKCε with other neuronal markers**. A. PKCε translocation (green) following exposure to thrombin (100 nM, 30 s) colocalises with other neuronal markers as shown. PKCε translocation was co-localised in c. half of cells with expression of the neuropeptide CGRP and with the neurofilament marker N52, and in a smaller proportion of cells with the neuropeptide substance P (SP) (panels on right). PKCε translocation was not in general co-localised with IB4 binding nor with parvalbumin (Prv) or COX-1 (panels on left). Neurones from adult mice cultured in the absence of NGF, with the exception of the COX-1 experiment which was carried out in neonatal rat sensory neurons cultured in NGF (100 ng/ml) as the antibody available to us did not bind mouse COX-1. Scale bars all 5 μm. B. Summary of results from experiments similar to those shown in A. First bar shows percentage of cells showing translocation of PKCε in response to thrombin (100 nM, 30 s). Remaining bars show percentages of these thrombin-responsive cells which co-expressed the neuronal markers noted beneath each bar. White bar in N52 column shows the proportion of the N52 positive neurons in which TRPV1 expression had been demonstrated by recording a calcium increase in response to capsaicin prior to fixation (c.f. Fig. 2). Final white bar shows overall fraction of thrombin-responsive neurons in which TRPV1 expression had been demonstrated by calcium imaging.

We next examined the histological characteristics of thrombin-responsive neurons, using translocation of PKCε as a marker. Around half of the thrombin-responsive neuronal population, predominantly medium-sized and large neurons, stained for neurofilament H (NFH^+^), a marker for myelinated neurons (second bar in Fig. 6B). Only a small fraction (around 6%) of these NFH^+ ^neurons also expressed functional TRPV1 receptors, as demonstrated by a calcium increase in response to application of capsaicin (see white bar at bottom of second bar in Fig. 6B), showing that this class of thrombin-responsive large neurons is predominantly non-nociceptive.

A distinct population of thrombin-responsive neurons, mainly small neurons, co-expressed the neuropeptides CGRP and/or substance P (bars 3 and 4 in Fig. 6B). Neurons in this population gave a calcium increase in response to capsaicin and therefore express TRPV1 (final bar in Fig. 6B). Very few thrombin-responsive small neurons were IB4-positive (5% of the thrombin-responsive population), in agreement with Fig. 2D above where neurons responding to thrombin with a calcium increase were found to be IB4-negative. The presence of neuropeptides and the lack of binding of IB4 identifies a TrkA positive C-fibre nociceptor sub-population [50] as the location of nociceptor PAR1/4 expression. In addition, thrombin-responsive neurons were negative for cyclooxygenase 1 (COX-1), an enzyme expressed in a subpopulation of small-sized nociceptive neurons [51], and for parvalbumin, expressed in non-nociceptive sensory neurons innervating muscle spindles [52]. In summary, our data show that functional receptors for thrombin are expressed broadly across all neuronal size classes, in neurons subtending both myelinated and unmyelinated fibres. In the unmyelinated neuronal population thrombin-responsive neurons are found in the peptidergic/IB4^- ^class of nociceptors.

### Release of CGRP by heat is potentiated by PAR1

**Figure 7:**
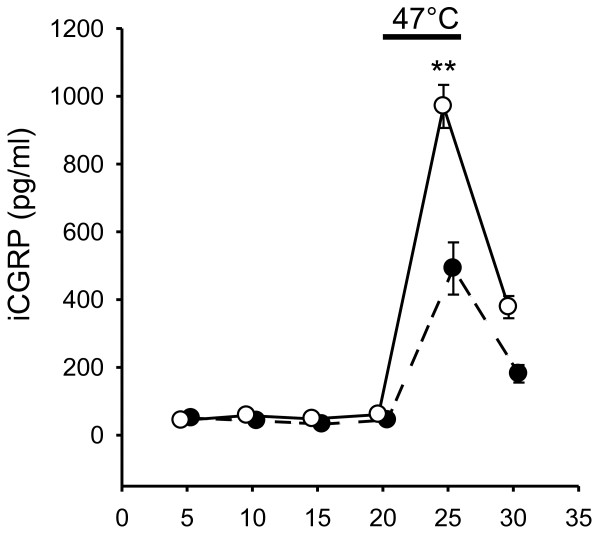
**Heat-induced CGRP release from isolated rat skin is facilitated by PAR1 activation**. A. CGRP release elicited by heat (closed circles, n = 16) was increased approximately two-fold by the rat PAR1-AP SFLLRN-OH (100 μM, open circles, applied during minutes 5 to 20, n = 12,). Points show mean ± SEM; **, p < 0.01 (ANOVA + Scheffé).

The results outlined above show that PAR1/4 receptors in small neurons co-express with TRPV1 and the neuropeptide CGRP, suggesting that neuropeptide release caused by TRPV1 activation should be potentiated by PAR1. Fig. 7 shows an experiment in which this hypothesis was tested using a rat skin preparation, which contains nerve terminals from which CGRP can be released by noxious heat stimulation [53]. In mouse skin, this heat response is markedly reduced, though not abolished, if the TRPV1 gene is deleted, and it is sensitized by pre-treatment with the weak TRPV1/2/3 agonist 2-APB which is ineffective in TRPV1 knockouts [54]. In the present experiments, the heat stimulation caused about a tenfold increase in CGRP release from cutaneous nerves (p < 0.001, t-test). The basal CGRP release was unaffected by the presence of PAR1-AP. The release in response to heat was approximately doubled by exposure to the PAR1-AP, consistent with expression of PAR1 in peptidergic neurons.

### Upregulation of PAR expression by neurotrophins

**Figure 8:**
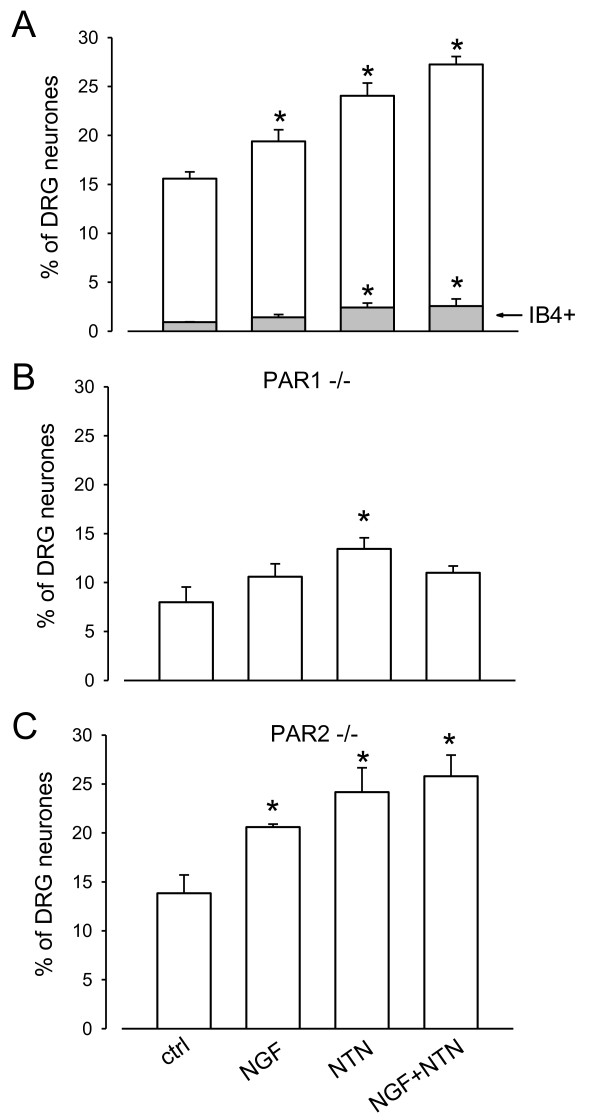
**Upregulation of proportion of thrombin-responsive cells by neurotrophic factors**. A. Percentage of neurons in which PKCε translocation was observed following exposure to thrombin (100 nM, 30 s) (white bars) was increased significantly by NGF (100 ng/ml, 3 days, p < 0.05) and by neurturin (NTN, 50 ng/ml, 3 days, P < 0.01). Effects were partially additive (final bar). Grey bars show percentage of neurons that were IB4^+^; neurturin caused significant upregulation of expression of thrombin responsiveness in the IB4^+ ^population but NGF had no significant effect. *; p < 0.05; **, p < 0.01 compared to control. B. Deletion of PAR1 reduces but does not eliminate responsiveness to thrombin. C. Deletion of PAR2 does not affect proportion of neurons responsive to thrombin.

Fig. 8 examines the effect of exposure to neurotrophic factors on expression of functional PAR1/4 receptors, measured from PKCε translocation following exposure to thrombin. NGF and neurturin (NTN) applied individually significantly increased the number of thrombin-responsive small neurons, while the effects of NGF and NTN applied together were additive, consistent with the known expression of TrkA and Ret receptors in separate neuronal populations (Fig. 8A). In the absence of neurotrophins few thrombin-responsive neurons bind IB4 (first bar in Fig. 8A). NGF increased the proportion of thrombin-responsive neurons but IB4 binding was not significantly increased. NTN, on the other hand, significantly upregulated the proportion of the thrombin-responsive population stained by IB4.

In neurons from PAR1^-/- ^animals the proportion showing PKCε translocation was significantly reduced but was not zero (Fig. 8B), consistent with an action of thrombin on PAR4 as discussed above. The fraction of responsive neurons was upregulated by NGF and NTN. In neurons from PAR2^-/- ^animals the proportion of neurons activated by thrombin, and the effects of NGF and NTN in upregulating the proportion of thrombin-responsive neurons, were similar to wild-type neurons (Fig. 8C), confirming that PAR2 receptors are not involved in responses to thrombin.

## Discussion

The work described here demonstrates a previously unsuspected role for PAR1 and PAR4 protease-activated receptors in nociceptive neurones. We used both histological and functional expression studies to explore the expression of PAR receptors in sensory neurons. For many of the functional studies we used thrombin, a protease which activates PAR1, 3 and 4 but not PAR2 receptors. In initial studies we used neurons from mice and rats, and from both adult and neonatal animals, in order to gain an understanding of how responses to PAR agonists differ across species and at different ages. In fact there were few significant differences (see Table 3) and we therefore focussed on adult mice in the majority of experiments in order to compare our results with those from PAR knockout animals.

**Table 3:** Summary of characteristics of DRG neurons from adult and neonatal mice and rats

	Adult mouse	Neonatal mouse	Adult rat	Neonatal rat
**% of THR-reponsive neurons (Ca imaging)**	17.5	15.2	14.5	19.2
**% of THR-reponsive neurons (PKCε translocation)**	19,4 ± 1,2	17,5 ± 1,5	13,4 ± 2,4	17,8 ± 1,0
**% of THR-responsive neurons which are IB4+**	0,9 ± 0,1%	1,0 ± 0,5%	0	0
**% of THR-responsive neurons responsive to PAR2-AP (Ca imaging)**	0	0	0	0
**THR sensitizes TRPV1 (Ca imaging)**	yes	yes	yes	yes
**PAR1-AP and PAR4-AP sensitize TRPV1 (patch clamp)**	yes	-	-	yes
**Segregation between PAR1 and PAR2 responsiveness (Ca imaging)**	yes	yes	yes	yes
				
**Neurons responsive to thrombin from day 1 in vitro**	yes	yes	yes	yes
**Upregulation by NTN of thrombin-responsiveness (PKCε translocation)**	yes	yes	yes	yes
**% of neurones responding to PAR2-AP expressing TRPV1 (Ca imaging)**	77,8 ± 9,4	91,0 ± 4,9	87,7 ± 6,2	88.6 ± 5.1%
**% of neurones responding to PAR2-AP which are IB4+ (Ca imaging)**	81.1 ± 8.3	81.8 ± 5,0	-	82.5 ± 6.0%
				
**% of THR-responsive non-neuronal cells in DRG cultures**	57,5 ± 5,6%	62,5 ± 9,9%	37,9 ± 7,0%	55,4 ± 3,4%

We find clear evidence for expression of PAR1/4 receptors in a population of peptide-expressing, IB4-negative nociceptive neurones, where they couple to PKCε, cause sensitization of TRPV1 and promote the heat-dependent release of the pro-inflammatory neuropeptide CGRP. These observations suggest a role for PAR1/4 receptors in promoting inflammation and pain following the release of thrombin. Functional PAR1/4 receptors are also found in a fration of large diameter neurones which express neurofilament H and would therefore in vivo subtend myelinated fibres. Only a small minority of these NFH^+ ^neurons express TRPV1 (Fig. 6B), suggesting that most serve a non-nociceptive function.

### PAR1

PAR 1 is expressed in around 15% of primary sensory neurons from adult mice. Consistent data were obtained from in situ hybridisation (15%, Fig. 1C), from immunohistochemistry (10%, Fig. 1D), in functional studies from sensitization of the capsaicin response (15%, Fig. 3G) and from translocation of PKCε (15.6%, Fig. 5B). These measures also showed that PAR1/4 expression was distributed approximately equally across all neuronal size classes (Fig. 1 and Fig. 5D). In agreement with this, approximately half of the neurons responding to thrombin were positive for neurofilament H, a marker for larger neurons subtending myelinated fibres (Fig. 6B). Most of the remainder of the thrombin-responsive neurons (c. one third of the total) were small and expressed functional TRPV1, TRPA1 and prokineticin and bradykinin B2 receptors (Fig. 2) and contained neuropeptides (Fig. 6B), all of which are characteristic of the small and medium-sized nociceptive neuronal population. The myelinated-fibre and nociceptor PAR1-expressing populations are mostly distinct, because only a small fraction of neurons expressing neurofilament H also express TRPV1 (Fig. 6B). Thus the PAR1-expressing neuronal population can be divided in broad terms into two functionally distinct classes: myelinated-fibre neurons, most of which do not express TRPV1; and unmyelinated-fibre neurons expressing neuropeptides, TRPV1 and other markers for nociceptors. Dai et al [16] found that PAR1 was not co-expressed with TRPV1 and therefore would not be expected to play a role in nociception, but in the present study we find by using several independent approaches that there is strong evidence for co-expression of functional PAR1 receptors and TRPV1 in the peptidergic subset of small and medium-sized neurones.

Several observations show that functional PAR1 and PAR4 receptors are not expressed in the non-peptidergic, IB4-positive class of nociceptors. Few thrombin-responsive neurons were IB4-positive (Fig. 2 and Fig. 6B), and small thrombin-responsive neurons express the neuropeptides CGRP and SP (Fig. 6) which are known not to be colocalized with IB4 binding. Finally, exposure to NGF increases the fraction of thrombin-responsive neurons (Fig. 8A), implying the presence of functional TrkA receptors, which are known to be expressed in the IB4^- ^population.

One important functional consequence of PAR1/4 activation in nociceptive neurons is that both receptors can sensitize the heat and capsaicin receptor, TRPV1, which in vivo has been shown to produce a state of heat hyperalgesia [55]. The membrane current carried by TRPV1 in response to either heat or capsaicin was approximately doubled in responsive neurons following exposure to thrombin or PAR1 and PAR4 activator peptides (Fig. 4). PAR receptors couple to G_q_, leading to activation of protein kinase C [5] which has in turn been shown to phosphorylate and sensitize TRPV1, as reviewed in [49]. Most of the sensitization of TRPV1 by PAR1 is abolished by PKC inhibitors (Fig. 3B), showing that phosphorylation by PKC is also the main pathway important in sensitization of TRPV1 by PAR1/4.

The sensitization of TRPV1 by thrombin, together with the observation that CGRP is expressed in the thrombin-responsive nociceptor population, suggests that the CGRP release activated by heat should be enhanced by PAR1 activation. This prediction was borne out in experiments performed on isolated rat skin containing intact peptidergic nerve terminals; the heat-dependent CGRP release was strongly potentiated by PAR1 activation (Fig. 6). The implication of this experiment is that PAR1 activation should play a role in potentiating neurogenic inflammation, in which neuropeptides such as CGRP are released from nociceptive nerve terminals following noxious insults or cell damage.

### PAR2

The role of PAR2 in sensory neurones has been explored fully in studies from other labs and was examined in less depth in the present study than that of PAR1/4. In situ hybridization (Fig. 1) showed that PAR2 is expressed almost exclusively in the small neuronal population, the majority of which are nociceptors, as was found by Amadesi et al [17]. In agreement with this, c. 80% of PAR2-AP responsive neurones expressed functional TRPV1 and TRPA1 ion channels (Fig. 2B). One surprise, though, in view of previous studies implicating PAR2 in neuropeptide release [13,15] was that the large majority of neurones in which PAR2-AP elicited a calcium signal were IB4-positive, and therefore belong to a population which is predominantly non-peptidergic (Fig. 2B).

### PAR3

PAR3 was strongly expressed, mainly in small neurones (Fig. 1). The percentages of neurones expressing PAR3 determined by in situ hybridization and immunohistochemistry were in good agreement (49% and 42%, respectively). However, when thrombin or trypsin, both of which activate PAR3 along with PAR1/4, were used in a number of different studies of functional expression, responses were seen in a significantly smaller proportion of neurones than those suggested by histological data for PAR3. Desensitization of PAR1/4, which should leave PAR3 unaffected, in fact largely ablated the response to thrombin (Fig. 3). Thus PAR3 must either be non-functional in sensory neurones, or else is only able to act in concert with other PAR receptors, as has been noted in other studies [see 47;48].

### PAR4

PAR4 expression, like PAR1, was found by in situ hybridization to be broadly distributed across all neuronal sizes (Fig. 1). There is clear evidence for a functional role for PAR4 in sensory neurons. PAR4-AP was found in patch clamp experiments to cause a sensitization of TRPV1 as potent as that of PAR1-AP, although in fewer neurons, and in calcium imaging experiments the percentage of cells sensitized by thrombin was reduced by less than half, from 15% to 8%, by genetic deletion of PAR1 (Fig. 4). Consistent with this, PAR1-AP caused translocation of PKCε in c. 15% of neurons, while PAR4-AP caused translocation in 9% (Fig. 5). PAR4 is expressed only in PAR1-expressing neurons, because all neurons responding to PAR4-AP also responded to PAR1-AP (Fig. 3). PAR2, by contrast, is expressed in a distinct neuronal subpopulation (Fig. 2B,D).

### Upregulation of PAR expression by neurotrophins

Neurotrophins enhance the sensation of pain partly by upregulating a wide variety of proteins important in nociception. We have shown that both NGF and neurturin upregulate thrombin-responsiveness in sensory neurones (Fig. 7). The increase in responsiveness was seen as an increase in the number of neurons expressing functional PAR1/4 receptors in response to a maximal dose of thrombin, suggesting the de novo appearance of functional receptors in neurons that previously did not express them, rather than sensitization of existing receptors. The action is on the small neurone population (Additional file 2), in agreement with the known expression of both TrkA and Ret in small nociceptive neurons. The results are consistent with an action of the two neurotrophins on separate neuronal populations, however, because NGF does not increase the few IB4^+ ^neurones which respond to thrombin, while NTN does increase the number of these IB4^+ ^neurons, consistent with the idea that NGF unregulates the number of PAR1/4 expressing neurons in the peptidergic population, while NTN induces de novo expression of PAR1/4 receptors in the IB4^+ ^neuronal population.

### Functional implications

Activation of PAR2 receptors is well known to cause inflammation [19] but a role for PAR1 and 4 is less clear. Previous studies have shown that PAR1/4 activation has a dual role: low doses are antinociceptive, while higher levels cause inflammation and pain [3,28,56,57]. The finding in the present study that PAR1/4 receptors are expressed in two distinct populations of sensory neurons suggests a possible basis for this dual effect. Activation of large-diameter myelinated afferents is well known to have an antinociceptive effect, and the activation of PAR1/4 in these afferents could therefore have an analgesic action. The expression of PAR1/4 in small-diameter nociceptive afferents, on the other hand, where they can potentiate TRPV1 and enhance the release of neuropeptides, provides a ready explanation for the inflammatory effects of higher levels of thrombin and specific PAR1/4 agonists. Following injury and rupture of blood vessels the release of significant amounts of thrombin could act on nociceptive nerve terminals, sensitizing TRPV1 to heat stimuli and promoting the release of pro-inflammatory neuropeptides such as CGRP, as has been shown in this study. Thus we propose that higher levels of thrombin can act in a similar way to other better-studied pro-inflammatory mediators, in promoting neurogenic inflammation and heat hyperalgesia in injured tissues through the sensitization of TRPV1.

## Conclusions

In summary, we find clear evidence for co-expression of functional PAR1 and PAR4 receptors in a sub-population of small peptide-expressing nociceptive neurones, where they couple to PKCε, cause sensitization of TRPV1 and promote the heat-dependent release of the pro-inflammatory neuropeptide CGRP. This study therefore suggests a previously unsuspected role for PAR1 and PAR4 in mediating the inflammation and pain caused by tissue damage severe enough to rupture blood vessels. Functional PAR1/4 receptors are also expressed in large diameter myelinated-fibre neurones which do not express TRPV1 and are therefore likely to be non-nociceptive. The role of PAR1/4 in these non-nociceptive neurones is less clear, but they may be responsible for the antinociceptive effects of low concentrations of thrombin.

## Competing interests

The authors declare that they have no competing interests.

## Authors' contributions

VV designed and carried out all experiments except those in Fig. 1 and Fig. 7, and wrote the first draft of the manuscript. AMK designed and carried out the histological experiments in Fig. 1, contributed to the Ca imaging experiments and commented on the manuscript. MP carried out calcium imaging and immunocytochemistry experiments on DRG cultures from transgenic animals, carried out data analysis and statistical analysis, participated in experimental design and in manuscript preparation. SH carried out the experiments in Fig. 7. PR designed the experiments in Fig. 7 and commented on the manuscript. PCM participated in experimental design and in manuscript preparation. CG participated in experimental work, equipment design and data analysis of electrophysiology experiments, carried out statistical analysis, wrote equipment software and image and data analysis software necessary for the work, and participated in manuscript preparation. PAM designed the project, advised on experiments, analyzed and interpreted data and wrote the final version of the manuscript, which was approved by all authors.

## Supplementary Material

Additional file 1 **Thrombin sensitizes TRPV1 in sensory neurons**. Click here for file

Additional file 2 **Upregulation by neurotrophic factors of PAR receptors**. Click here for file
