# Supplementary material for: Protease activated receptors 1 and 4 sensitize TRPV1 in nociceptive neurones
Source: Mol Pain. 2010 Sep 27;6:61. doi: 10.1186/1744-8069-6-61 (PMC2956715; doi:10.1186/1744-8069-6-61)
Supplement: Additional file 1 — Thrombin sensitizes TRPV1 in sensory neurons. [file 1744-8069-6-61-S1.DOC]

**Additional Figure 1** Thrombin sensitizes TRPV1 in sensory neurons.

A-C Distribution of ratios b/a (see Fig 3F) with control solution (A, n= 328 cells, 4 experiments), thrombin (B, n = 448, 5 experiments) and thrombin in PAR1^‑/-^ animals (C, n=348, 5 experiments). Cells sensitized by thrombin have ratios exceeding the 99.7% confidence limit obtained from the control distribution (arrow).
