# Supplementary material for: Protease activated receptors 1 and 4 sensitize TRPV1 in nociceptive neurones
Source: Mol Pain. 2010 Sep 27;6:61. doi: 10.1186/1744-8069-6-61 (PMC2956715; doi:10.1186/1744-8069-6-61)
Supplement: Additional file 2 — Upregulation by neurotrophic factors of PAR receptors. [file 1744-8069-6-61-S2.DOC]

**Additional Figure 2**. Upregulation by neurotrophic factors of PAR receptors activated by thrombin, as measured by translocation of PKCε. In presence of NGF (100ng/ml) and neurturin (50ng/ml, both 3 days) thrombin responsiveness is upregulated mainly in small DRG neurons - compare with Fig. 5E. Adult mouse neurons cultured in 10% FBS in absence of NGF and neurturin.
